# Supplementary material for: Distilling causality between physical activity traits and obesity via Mendelian randomization
Source: Commun Med (Lond). 2023 Nov 30;3:173. doi: 10.1038/s43856-023-00407-5 (PMC10689836; doi:10.1038/s43856-023-00407-5)
Supplement: Supplementary file 2 — Supplementary information [file 43856_2023_407_MOESM2_ESM.pdf]

SUPPLEMENTARY FIGURES

Supplementary Figure 1 – Agglomerative hierarchical clustering identifies five clusters of leisure screen time-associated loci ($P < 5x10^{-9}$) based on associations with BMI and years of schooling (**page 2**)

Supplementary Figure 2 – Mendelian randomization analyses between leisure screen time group 5 loci, years of schooling (schooling) and BMI (**page 3**)

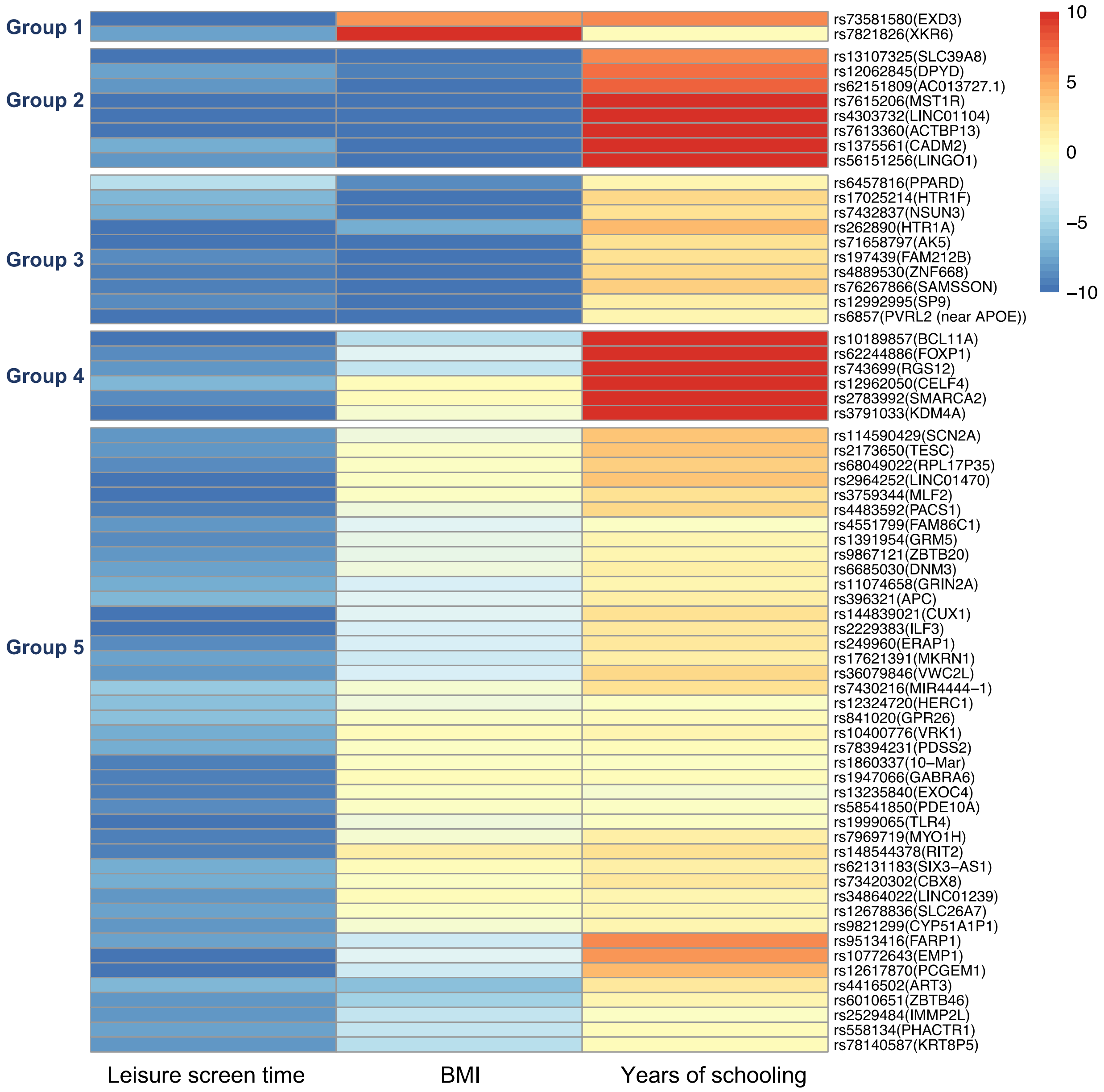

Supplementary Figure 1. Agglomerative hierarchical clustering identifies five clusters of leisure screen time-associated loci ($P < 5x10^{-9}$) based on associations with BMI and years of schooling. The color code illustrates the direction of association with the outcome (red = positive; blue = negative); and color intensity reflects the significance of the association (darker = lower P value) for 68 loci with summary statistics on all three traits available.

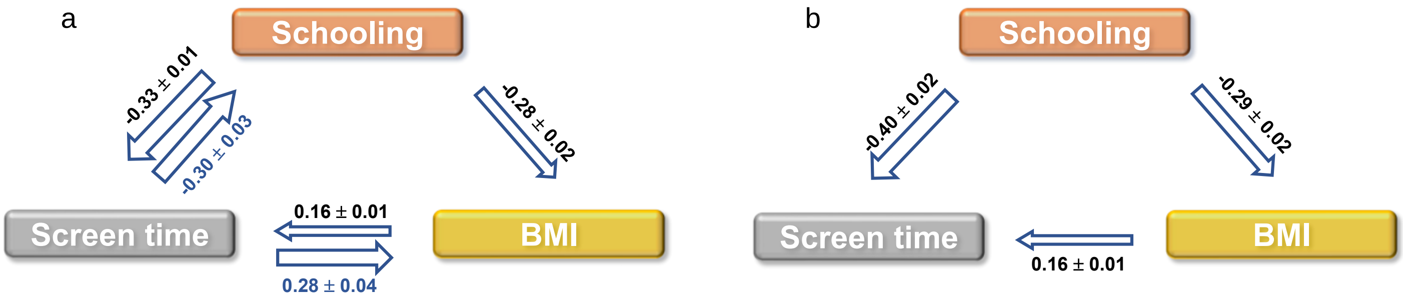

Supplementary Figure 2. Mendelian randomization analyses between leisure screen time (screen time) based on group 5 loci, years of schooling (schooling) and BMI. a, Causal estimates of univariable Mendelian randomization analyses using the MR-PRESSO method for screen time group 5 loci, i.e., loci mostly associated with screen time but not years of schooling and BMI. b, Causal estimates of Multivariable Mendelian randomization analyses using the IVW method for screen time group 5 loci. Results in black are not affected by the alternative instrumental variable for screen time and remain the same as in the main analyses (by design). Results are shown as beta ± SE: expressed in SD unit changes in outcome per 1 SD increase in exposure (original units: kg/m^2 for BMI, years for Schooling and hours/day for Screen time).
