# Supplementary material for: Distilling causality between physical activity traits and obesity via Mendelian randomization
Source: Commun Med (Lond). 2023 Nov 30;3:173. doi: 10.1038/s43856-023-00407-5 (PMC10689836; doi:10.1038/s43856-023-00407-5)
Supplement: Supplementary file 3 — Description of Additional Supplementary Files [file 43856_2023_407_MOESM3_ESM.pdf]

Description of Additional Supplementary Files

Supplementary Data 1. Genetic correlations of self-reported MV physical activity and screen time with years of schooling and obesity-related traits, acquired using LD score regression

Supplementary Data 2. List of Studies included in the two sample Mendelian Randomization analysis

Supplementary Data 3. Steiger outliers identified for each univariable MR test

Supplementary Data 4. Bidirectional causal inference of MV physical activity and BMI

Supplementary Data 5. Bidirectional causal inference of screen time and BMI

Supplementary Data 6. Bidirectional causal inference of MV physical activity and years of schooling

Supplementary Data 7. Bidirectional causal inference of screen time and years of schooling

Supplementary Data 8. Bidirectional causal inference from BMI to years of schooling

Supplementary Data 9. Total and direct causal effects estimated from univariable and multivariable Mendelian randomization (MR) analyses using inverse variance weighted (IVW) for MV physical activity

Supplementary Data 10. Identified MV physical activity and screen time loci and their associations with body mass index and years of schooling

Supplementary Data 11. Causal inference from screen time (group 5 loci only) to years of schooling

Supplementary Data 12. Causal inference from screen time (group 5 loci only) to BMI

Supplementary Data 13. Total and direct causal effect estimated from univariable and multivariable Mendelian randomization (MR) analyses using the inverse variance weighted (IVW) or MR-PRESSO method for screen time (group 5 loci only)
