# Supplementary material for: Distilling causality between physical activity traits and obesity via Mendelian randomization
Source: Commun Med (Lond). 2023 Nov 30;3:173. doi: 10.1038/s43856-023-00407-5 (PMC10689836; doi:10.1038/s43856-023-00407-5)
Supplement: Supplementary file 17 — Reporting Summary [file 43856_2023_407_MOESM17_ESM.pdf]

nature portfolio

Corresponding author(s): Zhe Wang, Marcel den Hoed

Last updated by author(s): May 7, 2023

Reporting Summary

Nature Portfolio wishes to improve the reproducibility of the work that we publish. This form provides structure for consistency and transparency in reporting. For further information on Nature Portfolio policies, see our Editorial Policies and the Editorial Policy Checklist.

Statistics

For all statistical analyses, confirm that the following items are present in the figure legend, table legend, main text, or Methods section.

n/a	Confirmed	
☐	☒	The exact sample size (*n*) for each experimental group/condition, given as a discrete number and unit of measurement
☐	☒	A statement on whether measurements were taken from distinct samples or whether the same sample was measured repeatedly
☐	☒	The statistical test(s) used AND whether they are one- or two-sided<br>*Only common tests should be described solely by name; describe more complex techniques in the Methods section.*
☒	☐	A description of all covariates tested
☐	☒	A description of any assumptions or corrections, such as tests of normality and adjustment for multiple comparisons
☐	☒	A full description of the statistical parameters including central tendency (e.g. means) or other basic estimates (e.g. regression coefficient) AND variation (e.g. standard deviation) or associated estimates of uncertainty (e.g. confidence intervals)
☐	☒	For null hypothesis testing, the test statistic (e.g. *F*, *t*, *r*) with confidence intervals, effect sizes, degrees of freedom and *P* value noted<br>*Give P values as exact values whenever suitable.*
☒	☐	For Bayesian analysis, information on the choice of priors and Markov chain Monte Carlo settings
☒	☐	For hierarchical and complex designs, identification of the appropriate level for tests and full reporting of outcomes
☐	☒	Estimates of effect sizes (e.g. Cohen's *d*, Pearson's *r*), indicating how they were calculated

Our web collection on statistics for biologists contains articles on many of the points above.

Software and code

Policy information about availability of computer code

Data collection	Publicly available GWAS summary statistics on GWAS Catalog and the IEU GWAS datasets were used
Data analysis	Mendelian randomization analyses were performed using the R packages TwoSampleMR (https://mrcieu.github.io/TwoSampleMR/), MR-PRESSO (https://github.com/rondolab/MR-PRESSO), cause (https://github.com/jean997/cause), lhcMR (https://github.com/LizaDarrous/lhcMR) and MVMR (https://github.com/WSpiller/MVMR).

For manuscripts utilizing custom algorithms or software that are central to the research but not yet described in published literature, software must be made available to editors and reviewers. We strongly encourage code deposition in a community repository (e.g. GitHub). See the Nature Portfolio guidelines for submitting code & software for further information.

Data

Policy information about availability of data

All manuscripts must include a data availability statement. This statement should provide the following information, where applicable:

- Accession codes, unique identifiers, or web links for publicly available datasets
- A description of any restrictions on data availability
- For clinical datasets or third party data, please ensure that the statement adheres to our policy

Our previously published GWAS summary statistics for physical activity and sedentary behavior is available on GWAS Catalog: https://www.ebi.ac.uk/gwas/publications/36071172

In this study we made use of data that is publicly available in the following platforms:
MR Base: https://www.mrbase.org/
IEU GWAS: https://gwas.mrcieu.ac.uk/

Human research participants

Policy information about studies involving human research participants and Sex and Gender in Research.

Reporting on sex and gender	GWAS summary statistics for both men and women were used in the analyses.
Population characteristics	Population characteristics of each study, for which we used GWAS summary statistics, can be found in their published manuscripts.
Recruitment	Not applicable
Ethics oversight	Not applicable

Note that full information on the approval of the study protocol must also be provided in the manuscript.

Field-specific reporting

Please select the one below that is the best fit for your research. If you are not sure, read the appropriate sections before making your selection.

☒ Life sciences ☐ Behavioural & social sciences ☐ Ecological, evolutionary & environmental sciences

For a reference copy of the document with all sections, see nature.com/documents/nr-reporting-summary-flat.pdf

Life sciences study design

All studies must disclose on these points even when the disclosure is negative.

Sample size	We used the GWAS results with the largest sample size available (physical activity and sedentary behavior: 661,399; BMI: 681,275; Years of Schooling: 766,345).
Data exclusions	No data were excluded
Replication	Replication of the primary MR results were conducted using the secondary analyses with a smaller list of instrumental variables, which strongly supported our main findings.
Randomization	Our approach (MR) is based on the quasi randomization of genetic variants.
Blinding	Not applicable

Reporting for specific materials, systems and methods

We require information from authors about some types of materials, experimental systems and methods used in many studies. Here, indicate whether each material, system or method listed is relevant to your study. If you are not sure if a list item applies to your research, read the appropriate section before selecting a response.

Materials & experimental systems

n/a	Involved in the study	
☒	☐	Antibodies
☒	☐	Eukaryotic cell lines
☒	☐	Palaeontology and archaeology
☒	☐	Animals and other organisms
☒	☐	Clinical data
☒	☐	Dual use research of concern

Methods

n/a	Involved in the study	
☒	☐	ChIP-seq
☒	☐	Flow cytometry
☒	☐	MRI-based neuroimaging
